# PCR-Based Direct Detection of *Streptococcus uberis* from Subclinical and Clinical Dairy Cattle Milk Samples

**DOI:** 10.1155/2020/8828624

**Published:** 2020-12-08

**Authors:** Virginia E. Sherwin, Morena Santi, Olivia Walker, Natalie D. Pickwell, Tracey J. Coffey, James A. Leigh, Sharon A. Egan

**Affiliations:** The School of Veterinary Medicine and Science, University of Nottingham, Sutton Bonington Campus, Sutton Bonington, Leicestershire LE12 5RD, UK

## Abstract

*Streptococcus uberis* is one of the leading causes worldwide of mastitis in the dairy industry, with the most likely sources of infection attributed to environmental reservoirs such as contaminated bedding materials. Early detection of those cases most likely to progress to clinical disease would lead to improved animal welfare, a critical component of overall health and productivity. A multiplex PCR-based diagnostic test was developed for detection of *S. uberis* directly from milk and targeting two genes previously identified as important for intramammary colonisation and persistence in dairy cattle. Results indicated the threshold for detection directly from milk was 20,000 CFU/ml and this was achieved without the need for preenrichment. In addition, *S. uberis* could be identified from milk samples collected during intramammary challenge studies, prior to clinical signs of infection and at much lower detection limits. The PCR test developed for confirmation of the presence of *S. uberis* directly from infected milk has potential value as a diagnostic test to identify early infection and/or to confirm that antibiotic therapy has been successful.

## 1. Introduction

Bovine mastitis is one of the most prevalent diseases within the dairy industry, resulting in significant economic and production losses, and is a considerable welfare issue for affected cows [1–3]. The current rate of clinical mastitis in the UK has been reported at 47 cases per 100 cows per year [4] with associated costs estimated to vary between £110 and £340 per cow [5–8]. There are an estimated 2.73 million dairy cows in the UK [9], and therefore approximately 1.28 million cases of clinical mastitis with financial losses attributed to vary between £141 and £436 million per annum in the UK alone. Confirmation of intramammary infection and early treatment of these animals are critical for reducing production costs and improving overall animal welfare. Treatment is most often initiated once clinical signs are apparent, with observed changes in the appearance, composition, and yield of milk and inflammation of the udder common [1]. It is estimated that, for control of intramammary infections in the UK dairy industry alone, 4 million tonnes of active ingredient is used each year [10]. Given the increasing threat of antimicrobial resistance [10], early diagnosis and targeted therapy for intramammary pathogens are critical, alongside monitoring treatment efficacy.

*Streptococcus uberis* (*S. uberis*) is an opportunistic, environmental dairy pathogen, responsible for 23% of clinical mastitis cases in the UK [4], with similar rates seen worldwide. It is ubiquitous within the dairy cow's environment and has been isolated from in and on the dairy cow, including the skin, rumen, genitourinary tract and faeces, bedding, and pasture [11–14]. Clinical mastitis due to *S. uberis* typically corresponds with high bacterial numbers between 10^6^ and 10^8^ cfu/ml of milk [15, 16] although bacterial concentrations may peak as high as 10^9^ cfu/ml. Chronic subclinical infection has been attributed to *S. uberis* where it may act as a contagious source of the pathogen [17, 18]. Confirmation of *S. uberis* as the causative bacterial agent forms an integral part herd management for mastitis control and this can influence antimicrobial therapy choices for treatment and whether drying off or culling may be required. In the majority of on-farm cases, animals are treated with broad-spectrum antibiotics upon signs of clinical infection, with no specific determination of the species responsible.

The accepted “gold standard” for diagnosis of *S. uberis* infection is following direct culture of milk on blood aesculin plates, with identification in most clinical laboratories utilising biochemical testing or API-based strip tests or enzymatic profiling [19]. Whilst advantages include the detection of viable bacteria, and the relatively inexpensive nature of culture based diagnostics, characterization based on phenotypic markers can be unreliable due to a lack of unique biochemical markers present among mastitis pathogens, including streptococcal and enterococcal species [20–23]. The time taken for culture-based confirmation, typically 2–3 days after recognition of clinical signs of infection, alongside problems with nonculturable organisms from milk samples means that this diagnostic approach is undersubscribed for infection management [4, 24].

The use of DNA-based diagnostic tests can allow rapid screening of large numbers of samples and have the potential to be extremely specific and allow distinction of closely related organisms, given careful design of the target sequence. They have also been previously used to detect bacteria in clinical samples which failed to grow in culture, as they are not dependent on the presence of viable bacteria within samples [22, 25, 26]. PCR-based assays confer an advantage of rapid turnaround time and can eliminate the subjectivity of assay interpretation, with sensitivity of PCR greater than traditional culture-based methods [27, 28]. Several studies have evaluated PCR for detection of *S. uberis*; however, specificity and sensitivity are often lower in comparison with other common mastitis pathogens and may reflect the choice of genomic target for the diagnostic PCR tests that have been previously developed [22, 26, 28–30]. Food products such as milk also contain PCR inhibitors including fat, protein, and calcium, and DNA extraction prior to testing is often key for a successful diagnostic outcome [31, 32]. Bacterial DNA can be readily extracted using organic solvents such as phenol-chloroform or using salt precipitation to remove such contaminants, providing a relatively cheap and cost-effective method for DNA isolation in comparison with commercially available kits [33, 34].

Here, we describe the evaluation of a multiplex PCR-based test for identification of *S. uberis* directly from milk, utilising highly conserved gene targets. The ability to detect the presence of *S. uberis* at levels below those associated with clinical mastitis indicated the potential use of this assay for early detection of infection or confirmation of successful therapeutic treatment.

## 2. Materials and Methods

### 2.1. Bacterial Strains and Reagents

*S. uberis* strain 0140 J (strain ATCC BAA-854/0140 J), originally isolated from a clinical case of bovine mastitis in the UK, and 12 other previously characterised clinical strains [35] were used throughout this study. The *Escherichia coli* strain P4 and *Staphylococcus aureus* strain M60 were both similarly isolated from clinical cases of bovine mastitis [36, 37]. *S. agalactiae* stains 0247a and 0251 and *S. dysgalactiae* 0154 and A1 were obtained from historical cases of mastitis in the UK. *L. garvieae* 131016 was isolated from a case of clinical mastitis in the UK and provided by Professor A Bradley (University of Nottingham). *E. faecium* 7831 was provided by Professor P. Barrow (University of Nottingham). *S. pneumoniae* TIGR4 [38] was included as a nonmastitis pathogen control. A total of 32 mastitic milk samples were collected from a 230-dairy-cow herd in Leicestershire, UK, with a history of environmental mastitis and identified through national milk record (NMR) testing. Milk samples were cultured on sheep blood agar containing 1% (w/v) aesculin (Cherwell Laboratories). All bacterial strains were confirmed to be the correct species by 16 S rDNA-based sequencing. Bacteria were routinely grown in Todd Hewitt (THB) or Brain Heart Infusion (BHI) broth (Oxoid Ltd, UK) at 37°C.

### 2.2. DNA Extraction

*S. uberis* 0140 J was spiked into 1 ml of whole milk and serially diluted 1 : 10, to provide a total of 9 spiked milk samples and 1 milk-only control. Three DNA extraction methods were evaluated to compare their relative efficiency with respect to the extraction of *S. uberis* DNA from milk: an in house phenol : chloroform based extraction, the PowerFood Microbial DNA Isolation kit (Qiagen) and QuickExtract DNA extraction solution (Epicentre). The phenol extraction protocol was performed as previously described [15]; however, bacterial cell walls were disrupted with cell disruption buffer containing 60 units/mL mutanolysin and 20 mg/mL lysozyme (both from Sigma-Aldrich, UK) and total cell lysis was achieved using 50 *μ*L of SDS solution (20% w/v in 50 mM Tris-Cl, 20 mM EDTA, pH 7.8) and 200 *μ*g/mL proteinase K (Sigma). Isolation of DNA using the Power Food Microbial DNA Isolation kit was performed as per manufacturers' instructions, using the alternative lysis method when there is difficulty in lysing cells. Isolation of DNA using the QuickExtract DNA solution was performed after centrifugation of samples at 13,000 ×g for 5 min and resuspension of the resulting pellet in 500 *μ*L of QuickExtract solution. The solution was vortexed for 15 sec, heated at 65°C for 15 min, vortexed for 15 sec then heated at 98°C for 2 min as per manufacturer's instructions, and stored at −20°C prior to use. All other bacterial DNA samples were generated from 1.5 ml of overnight culture using the standard phenol : chloroform extraction protocol previously described [15].

### 2.3. Primer Design for Identification of *sub0888* and *sub1154*

Primers were designed to amplify conserved regions of *sub0888* and *sub1154*, both previously identified as unique to *S. uberis* [39] and, in the case of *sub1154*, essential clinical mastitis following experimental challenge [15]. Sequences were analysed for conservation and identity within the NCBI nonredundant nucleotide basic local alignment database [40] and the *S. uberis* MLST database (https://pubmlst.org/suberis/) [41, 42].

### 2.4. PCR Amplification of Target Genes

PCR amplification of 16 S rDNA was performed using *Streptococcus* specific primers (Table 1) [43] and GoTaq Green Master Mix (Promega) containing 0.5 *μ*M of each primer and approx. 5 ng of DNA template. PCR amplification was performed using a LifeEco thermal cycler (BioER) with amplification programme consisting of an initial denaturation step at 95°C for 2 min, followed by 30 cycles, denaturation at 95°C for 20 sec, annealing at 60°C for 30 sec, and extension at 72°C for 45 sec with a final elongation step at 72°C for 5 min. PCR amplification of *sub0888* and *sub1154* was performed using GoTaq Green Master Mix (Promega) as above, using primer concentrations of 0.5 *μ*M for P1100 and 1101 and 0.25 *μ*M for P1102 and 1103 (Table 1) and an annealing temperature of 61°C. Quality and quantity of the PCR product were assessed following separation by 1% agarose gel electrophoresis and PCR products were purified using MinElute gel extraction (Qiagen) according to manufacturer's instructions. Quantification was performed using Qubit™ dsDNA BR assays (Thermo Fisher) and sequencing of the purified product was performed by Source Biosciences using P665 as the sequencing primer.

### 2.5. Cattle Challenge Experiments

Frozen milk samples from previous dairy challenge experiments [15, 44] conducted at IAH, Compton Laboratory under PPL 30/2645, were used in this study to determine the detection limits for *S. uberis* 0140 J within milk. Briefly, 4 Holstein–Friesian cows, 2–10 weeks into their first lactation with no previous history of mastitis, were challenged with approximately 1000 cfu/ml of *S. uberis* 0140 J in two contralateral quarters. Animals were milked and inspected twice daily (07 : 00 h and 15 : 30 h) and treated with appropriate antibiotics once clinical end points had been reached using criteria previously described [15, 45] and clinical scores recorded for changes in milk and udder quarters. Milk samples were taken at each milking and analysed for the presence of bacteria and somatic cells, with viable bacteria estimated by direct plating of each milk sample onto aesculin blood agar plates.

## 3. Results

### 3.1. Generation of *sub0888* and *sub1154* PCR Primers for Identification of *S. uberis*

Primers were designed to amplify a conserved region of DNA, 222 bp upstream and then internal to *sub0888* of *S. uberis* 0140 J to produce a product of 974 bp. Primer sequences showed 100% identity with sequences in 3 additional completed *S. uberis* genome sequences (NCTC4674, NCTC3858, and NZ01) and 12 partially completed genomes [35]. Additionally, the sense primer P1100 showed 100% identity to all strains within the *S. uberis* BIGSdb database and the antisense primer 82% identity, with a single base mismatch identified within the remaining strains at position 15 (G : A) for 21 strains and position 9 (T : A) for a further 2 strains. Of the total 132 strains in the MLST database, one gene sequence was truncated for *sub0888* and this sequence was incomplete. Primers designed to amplify an internal conserved region of *sub1154* produced a product of 573 bp and showed 100% identity to all known *S. uberis* sequences on both the NCBI and MLST databases.

### 3.2. Comparison of Multiplex Primers for *S. uberis* Detection

**Figure 1:**
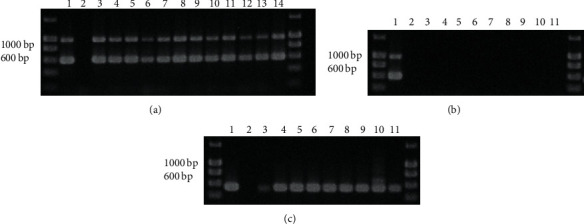
Multiplex PCR for *S. uberis* detection. (a) Detection of *sub0888* and *sub1154* in *S. uberis* strains EF20, 6736, 6780, Ab71, B190, B362, C5072, C5388, C6344, C8329, C9359, S6261 (lanes 3–14), *S. uberis* 0140 J and no template control in lanes 1 and 2, respectively. (b) Detection of *sub0888* and *sub1154* in other bacterial strains, *E. coli* P4, *S. aureus* M60, *S. agalactiae* 0247a and 0251, *S. dysgalactiae* 0154 and A1, *L. garvieae* 131016 (isolated from cases of clinical mastitis), *E. faecium* and *S. pneumoniae* TIGR4 in lanes 3–11, respectively, *S. uberis* 0140 J and no template control in lanes 1 and 2, respectively. (c) Confirmation of presence of 16 s rRNA in other bacterial strains, as analysed in Figure 1(b).

Genomic DNA was extracted from 12 strains of *S. uberis* and presence of *sub0888* and *sub1154* confirmed by multiplex PCR (Figure 1(a)). In addition, the multiplex PCR was performed using genomic DNA from a number of bacterial pathogens previously associated with mastitis including *E. coli*, *S. aureus*, *S. agalactiae*, *S. dysgalactiae*, and *L. garvieae* and a number of related species from the Streptococcaceae family (*E. faecium* and *S. pneumoniae*), all of which were negative (Figure 1(b)), with *16* *S rDNA* PCR performed to confirm the presence of genomic DNA in these samples (Figure 1(c)).

### 3.3. Comparison of DNA Extraction Methods from Milk for *S. uberis* Detection

**Figure 2:**
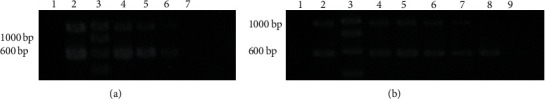
Comparison of DNA extraction methods from milk. Detection of sub0888 and sub1154 in *S. uberis* 0140 J spiked milk from (a) PowerFood Microbial DNA Isolation kit detected to a level of 2 × 10^6^ cfu/ml and (b) phenol : chloroform extraction detected to a level of 2 × 10^4^ cfu/ml.

Whole milk (1 ml) was inoculated with approx. 2 × 10^8^ cfu of *S. uberis* and serially diluted 10-fold to a concentration equivalent to ∼2 cfu/ml. DNA was extracted from the samples using either an in-house phenol : chloroform extraction method, the PowerFood Microbial DNA Isolation kit (Qiagen), or the QuickExtract DNA Extraction solution (Epicentre). Each PCR was performed using 1 *μ*L of extracted DNA using the GoTaq G2 Hot Start Green Master Mix. Bacteria were detected at a concentration of 2 × 10^6^ cfu/ml in spiked milk processed using the PowerFood Microbial DNA Isolation kit and 2 × 10^4^ cfu/ml in milk processed using the phenol : chloroform extracted DNA (Figure 2). No amplification of DNA was detected in samples that were extracted using the QuickExtract DNA extraction solution. In addition, DNA was unable to be amplified using either Standard Taq (with ThermoPol buffer) or Phusion polymerases (NEB) (data not shown) using DNA isolated from the spiked milk samples.

### 3.4. Recovery and Confirmation of *S. uberis* Strain Type from Clinical Mastitis Samples

**Figure 3:**
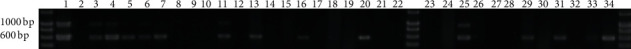
Multiplex PCR analysis for clinical mastitic milk samples. Detection of *sub0888* and *sub1154* directly from mastitic milk samples with 14 of 32 samples identified as positive. Smearing correlated with samples that had large cell pellets, most likely due to high numbers of somatic cells within the samples. *S. uberis* 0140 J positive control and negative control are in lanes 1 and 2, respectively.

Frozen milk samples (*n* = 32) were provided by a local Leicestershire farm, with a history of environmental clinical mastitis. Milk samples were thawed and plated onto ABA plates, with single colonies selected for growth and DNA extraction. Bacterial identity was confirmed for 50% of the samples by 16 S rDNA-based sequencing which identified 13 as *S. uberis*, 2 as *Staphylococcus sciuri*, and 1 as *Bacillus licheniformis*. Each milk sample (1 ml) was processed to obtain DNA using the phenol : chloroform extraction method and 14 samples were identified as positive for *S. uberis* based on the multiplex PCR (Figure 3), including all those identified as *S. uberis* by 16 S rDNA sequencing. One additional milk sample was found to be positive by PCR and bacterial culture was unable to be obtained from this sample.

### 3.5. Recovery of *S. uberis* 0140 J as Assessed by PCR from Experimental Challenge Samples

**Figure 4:**
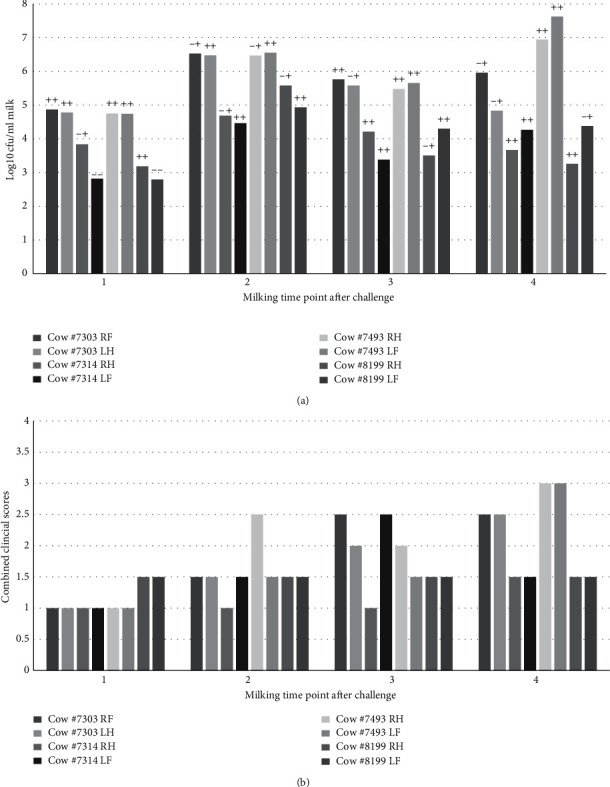
Detection of *S. uberis* from experimentally challenged cows in comparison to bacterial concentration and clinical score. (a) Comparison of bacterial cell counts obtained from direct plating of infected milk samples and detection of *S. uberis* from the same samples by multiplex PCR. (--) negative for sub0888 and sub1154, (−+) positive for sub1154 only, (++) positive for sub0888 and sub1154, with bacterial detection by PCR confirmed by 24 hrs after infection in all samples. (b) Clinical scores for milk quality and composition as per previous published guidelines (Field 2003).

DNA was extracted from 1 ml of milk using the phenol: chloroform extraction method from quarter milk samples obtained from 4 cattle challenged in two contralateral quarters with *S. uberis* 0140 J [15, 44]. *S. uberis* was detected by multiplex PCR in 75% of samples obtained after 16 hours of challenge and 100% after 24 hours of challenge (Figure 4(a)). This corresponded with a bacterial concentration of greater than 1000 cfu/per ml of milk. Bacterial presence was confirmed to occur prior to observation of clinical signs (Figure 4(b)), as outlined in previous studies [45].

## 4. Discussion

The successful implementation of the “Five-Point Plan” [46] has significantly reduced the rates of clinical mastitis associated with contagious bacterial pathogens *Staphylococcus aureus* and *Streptococcus agalactiae*. Given the difficulty in controlling environmental mastitis pathogens particularly those identified within the faecal microbiota such as *E. coli* and *S. uberis*, it is not surprising that rates of clinical mastitis associated with these pathogens have continued to rise [4, 47]. Management practices should also include environmental management of pasture, bedding, and housing to minimise faecal contamination risks. In addition, the development of rapid, low-cost, species-specific diagnostic tests for targeted antimicrobial therapy is pivotal for identifying early clinical cases of *S. uberis* infection and to confirm that antibiotic therapy has been successful. This study focused on the development of a multiplex PCR diagnostic test for the detection of *S. uberis* DNA directly from milk samples, using highly conserved genomic sequences, and was able to confirm the presence of *S. uberis* within milk obtained from naturally and experimentally infected animals.

PCR-based assays have been investigated as an alternative to traditional culture-based methods, for a many different mastitis pathogens, including *S. uberis*. Primarily, this is due to the rapid results that can be obtained in comparison to culture-based assessment and subsequent biochemical testing, which generally takes 2–3 days to yield a specific bacterial identification. An additional benefit is that identification of nonculturable organisms can be achieved, particularly important for subclinical mastitis samples when bacterial numbers may fluctuate [4, 24]. Studies have evaluated PCR as a viable method for *S. uberis* detection, each with variable gene target choices, where target information has not been specified [22, 26, 28–30], or have focused on amplification of specific 16 S and 23 S rDNA regions, which in some cases made differentiation of amplified products difficult in post-PCR analysis [22, 23, 31].

Given the rapid increase in available genome sequences for comparative analysis of bacterial isolates, primers in this study were designed to target *sub0888* and *sub1154* of *S. uberis*, both previously identified as sortase anchored, surface anchored proteins [48]. It was decided to use *sub0888* as a target as it has been identified to share no known homology with genes in any other bacterial genome and appears to be unique to *S. uberis*. The second target, *sub1154*, shows very low (<30%) identity to the C5a peptidase of *S. pyogenes* and a number of other streptococcal proteins [39, 48]. The resulting protein is essential for early colonisation of the udder and subsequent infection during clinical mastitis [15]. The specific primer sequences used in this study were selected to conserve DNA sequences present, known to be present in all annotated *S. uberis* genomes, present on NCBI genome databases [40]. Further analysis of sequence conservation was conducted on sequences present in *S. uberis* MLST database, with 100% identity for the *sub1154* primers and for the *sub0888* primers and 99.2% identity when allowing for a 1 base pair mismatch [49, 50], and would therefore provide high specificity for *S. uberis* within the PCR assay. The *S. uberis* strains within both databases include clinical and subclinical isolates from the UK, Canada, and New Zealand and it is reasonable that this would be similar for other strains isolated around the world. Given the sequence conservation of the primers used in the multiplex PCR, it was not surprising that amplification using DNA extracted from bacterial culture successfully amplified both genes in a range of *S. uberis* strains (Figure 1(a)) and additionally did not result in amplification of products from a range of related streptococci or mastitis-related pathogens (Figure 1(b)).

Whilst a number of PCR-based tests have been assessed including the PathoProof diagnostic test, which tests for the 12 main causative pathogens associated with mastitis, lower sensitivity and specificity for *S. uberis* detection have been reported compared with other mastitis pathogens such as *S. aureus* [28]. This may also be related to difficulty in extracting high-quality DNA from some Gram-positive bacteria, in particular when using commercially available DNA extraction kits and isolation directly from milk [31, 51] or variability in the target genomic sequence selected for PCR analysis. For any PCR-based analysis, the quality of the DNA and removal of any inhibitory components which may be present in milk is critical for reproducibility of the assay. Numerous methods for extracting bacterial DNA directly from milk have been reported in literature including the use commercially available kits such as the bacterial DNA isolation kit from Norgen Biotek and the DNeasy PowerFood Microbial Kit from Qiagen, magnetic beads to bind DNA, alkaline or detergent extraction [52], pronase [30], and phenol-chloroform based extraction [53]. Comparative studies indicated the Qiagen DNeasy PowerFood microbial kit and Novogen Milk Bacterial Isolation kit could provide high yields of Gram-positive bacterial DNA; however, PowerFood kit was superior for DNA purity [51, 54]. This kit was selected for comparison, alongside an in-house, phenol-chloroform based method [55]. A key component of the in-house lysis method utilises the enzyme mutanolysin from *Streptomyces globisporus* which has been found to be superior for lysis of some Gram-positive bacterial species, particularly streptococci, in comparison to other enzymes such as lysozyme [56, 57]. In this study, phenol-chloroform-based exaction was superior to that of the commercial kit and could detect *S. uberis* at a lower limit of 1 × 10^4^ cfu/mL in spiked milk studies (Figure 2) and supports previous studies which reported to be a successful mechanism for removal of inhibitory substances for downstream PCR-based applications [58]. Similar detection limits were observed when tested on milk samples from experimentally challenged animals (Figure 4), and these could confirm the presence of *S. uberis* prior to observation of clinical changes in milk or udder.

Whilst detection at levels of between 10^4^ and 10^5^ cfu/ml may seem relatively high compared to some of the more sensitive RT-PCR methods, previous experimental challenge studies have indicated that this bacterial concentration in milk results in a “tipping point” where animals are highly likely to progress to clinical mastitis requiring antibiotic therapy [15, 44, 45, 59]. Given this, detection of *S. uberis* at a level lower than 1000 cfu/ml may not be clinically relevant and result in overtreatment of animals unlikely to progress to clinical disease. One surprising aspect was the different performance of the primers on DNA extracted from clinical samples, in particular when the bacterial cell counts were between 1 × 10^4^ and 1 × 10^5^ cfu/ml. In these cases the *sub1154* primers performed more reliably than the *sub0888* primers, potentially due to the smaller product produced in the assay as the primer melting temperature for both primers was similar. The highly conserved nature of both *sub0888* and *sub1154* within isolates indicates that each target could be suitable for future qRT-PCR development; however, the likely increased sensitivity would need to be balanced, given that detection of low numbers of *S. uberis* may not correlate to progression to clinical mastitis.

Overall, this DNA extraction and PCR application for detection of *S. uberis* directly from milk provides a reproducible method for diagnosis of cattle likely to progress to clinical mastitis, using highly specific gene targets. The implementation of such a diagnostic could form part of a herd management strategy, to identify cases of infection most likely to progress to clinical mastitis, before the absence of overt clinical signs. In addition, it could be used to confirm successful antibiotic therapy for *S. uberis* mastitis, leading to improved animal health, welfare, and therapeutic outcome.

## Figures and Tables

**Table 1:** Oligonucleotide primer sequences used in this study.

Designation	Target	Primer sequence	Product size	Annealing temp (°C)
665 Fwd 666 rev	16 S rRNA 16 S rRNA	5′-GAGAGTTTGATCCTGGCTCAGGA-3′ 5′-TTACCGCGGCTGCTGGCACGT-3′	529 bp	60
1100 Fwd 1101 rev	*S. uberis sub0888* *S. uberis sub0888*	5′-CTTTATGAAAATAGCCAAGCTGAAA-3′ 5′-TGTGAGCCAGTTGGAGGAAG-3′	974 bp	61
1102 Fwd 1103 rev	*S. uberis sub1154* *S. uberis sub1154*	5′-ACAAAGTTGAAAAGGGGCGT-3′ 5′-CGCCATTAGGTGAAAGTGCT-3′	573 bp	61

## Data Availability

Data associated with this manuscript are available upon request to Dr. Sharon Egan.
